# Ykl-40 and cancer antigen 72-4 as new and promising diagnostic and prognostic markers for endometrial cancer

**DOI:** 10.4274/tjod.77906

**Published:** 2019-01-09

**Authors:** Suat Karataş, Veysel Şal, İlker Kahramanoğlu, Fuat Demirkıran, Tugan Beşe, Macit Arvas, Nigar Sofiyeva, Onur Güralp, Hafize Uzun

**Affiliations:** 1 İstanbul Şişli Hamidiye Etfal Training and Research Hospital, Clinic of Obstetrics and Gynecology, İstanbul, Turkey; 2 İstanbul University Cerrahpaşa Faculty of Medicine, Department of Obstetrics and Gynecology, Division of Gynecologic Oncology, İstanbul, Turkey; 3 Yale University Faculty of Medicine, Department of Obstetrics and Gynecology and Reproductive Sciences, New Haven, CT, USA; 4 Klinikum Oldenburg University Hospital, Clinic of Obstetrics and Gynecology, Oldenburg, Germany; 5 İstanbul University Cerrahpaşa Faculty of Medicine, Department of Biochemistry, İstanbul,Turkey

**Keywords:** YKL-40, cancer antigen 72-4, endometrial cancer, screening

## Abstract

**Objective:**

To determine the predictive role of serum levels of YKL-40 and cancer antigen (CA) 72-4 in the diagnosis of endometrial cancer (EC).

**Materials and Methods:**

Forty-one patients with EC and 21 women with uterine polyps were evaluated between January and December 2015 in a prospective study.

**Results:**

Age, body mass index, preoperative serum YKL-40 and CA 72-4 levels were significantly higher in the malignant group compared with the control group. Serum YKL-40 levels were significantly higher in patients with superficial myometrial invasion and no lymph node involvement (p=0.042; p=0.004). No relationship between clinicopathologic factors and serum CA 72-4 levels was found.

**Conclusion:**

Serum CA 72-4 and YKL-40 levels are increased in women with EC compared with uterine polyps. Preoperative serum YKL-40 levels may be associated with favorable prognostic factors. The determination of YKL-40 before surgery may be helpful in the evaluation of the regional lymph nodes.

**PRECIS:** Serum YKL-40 and CA 72-4 may be used in the prediction of endometrial cancer.

## Introduction

Abnormal uterine bleeding is the initial symptom in 75 to 90% of the patients with endometrial carcinoma (EC). Most EC (80%) is diagnosed at stage 1 and five-year survival rates exceed 95%. A significant portion of patients have recurrent or advanced disease at admission and outcome is poor in these patients. In patients with regional spread, five-year survival rates are about 68% and if there is distant disease, five-year survival rates drop to 17%^(1)^.

In recent years, tumor markers have been widely investigated in order to detect EC during early phases and to monitor the disease. Numerous tumor markers have been studied for this purpose. Nevertheless, there are currently no markers routinely used in the diagnosis of the EC. Cancer antigen (CA)-125 has been reported to be high in 19% to 40% of patients with EC^(2,3)^. Sood et al.^(4) ^found that high serum CA-125 levels were strongly associated with the prediction of extrauterine disease and high mortality. However, CA-125 values do not seem to be useful in the early diagnosis of the EC. Serum human epididymis protein (HE)-4 levels were significantly higher in patients with recurrent EC. Furthermore, HE-4 was likely to be superior to CA-125 in detecting recurrent EC^(5)^. Both of these markers are significantly correlated with higher histologic grade, stage, lymph node metastases, myometrial invasion, and cervical involvement in patients with EC^(6,7,8)^.YKL-40 (Human Chitinase-3-like protein 1) is a glycoprotein that belongs to the chitinase family. Its exact function is not yet clearly known. High serum levels of YKL-40 are associated with extracellular matrix breakdown and angiogenesis^(9)^. Elevated serum YKL-40 levels have been reported in certain cancer types such as breast cancer, colorectal cancer, lung cancer, glioma, leukemia, melanoma, and some diseases including hepatic fibrosis, osteoarthritis and rheumatoid arthritis^(10,11,12,13,14,15,16,17)^. CA 72-4, a human tumor-associated glycoprotein (TAG), is frequently used as a tumor marker for diagnosing and predicting prognosis in gastric and ovarian cancers^(18,19)^. CA72-4 TAG-72 is not affected by pregnancy or the menstrual cycle phase, and it is barely influenced by inflammatory conditions^(19,20)^. In this study, our objective was to determine the efficacy of YKL-40 and CA72-4 in the early diagnosis of EC and to evaluate whether both markers had prognostic value for EC.

## Materials and Methods

### Study population

The study was performed in İstanbul University Cerrahpaşa Faculty of Medicine, Department of Gynecology and Obstetrics, Division of Gynecologic Oncology between January and December 2015. The study was approved by the Ethics Committee of İstanbul University Cerrahpaşa Faculty of Medicine (approval number: 83045809/604.01/02-46067). Approval of the local ethics committee was provided and the study protocol adhered to the principles of the Declaration of Helsinki. Informed written consent for participation in the study was obtained from all women. The manuscript was prepared in accordance with the Strengthening the Reporting of Observational Studies in Epidemiology statement^(21)^.The exclusion criteria were defined as presence of one of the following conditions: (i) a suspicious secondary malignancy, (ii) systemic disease such as renal and/or hepatic failure, congestive heart failure, chronic respiratory disease, (iii) neoadjuvant chemotherapy, (iv) history of chemotherapy or radiotherapy for any malignancy, (v) history of endometriosis, and (vi) major intraperitoneal disease (e.g., Crohn’s disease, ulcerative colitis).The inclusion criteria in the study and control groups were as follows: 27 women with menometrorrhagia or postmenopausal bleeding who underwent saline infusion sonography and had a pre-diagnosis of endometrial polyp in our clinic (internal) were enrolled. Blood samples were taken shortly before the endometrial biopsy in all women with the pre-diagnosis endometrial polyps. The histologic diagnosis was confirmed as endometrial polyps in 21 women, and these patients made-up our control group. Two women had proliferative endometrium and four women had EC. The women with EC were included in the study group. All patients with endometrial polyps underwent hysteroscopic resection. Thirty-seven women who underwent endometrial biopsy in an external center and were diagnosed as having EC were enrolled in the study. Blood samples were taken shortly before surgery in all women with a diagnosis of EC. The laparoscopic or laparotomic operations were performed in our clinic. Surgical staging included total hysterectomy, bilateral salpingo-oophorectomy, pelvic lymphadenectomy, and para-aortic lymphadenectomy, if necessary. A total of 41 women (4 internal + 37 external) with EC and 21 women with endometrial polyps were included in the statistical analysis.

All histopathologic diagnoses were made by two gynecologic pathologists. Histologic type and stage of the disease according to International Federation of Gynaecology and Obstetrics were available in all patients with EC^(22)^. The charts and pathologic findings were reviewed in a blinded fashion, without knowing the preoperative serum YKL-40 and CA 72-4 values.

### Biochemical analysis

Blood samples were collected in ethylenediamine tetraacetic acid (EDTA)-containing tubes and anticoagulant-free tubes after an overnight fast. Plasma and serum were separated immediately and stored at -80 °C until required for analysis.

### Measurement of serum YKL-40 concentrations

Serum YKL-40 concentrations were determined using a commercial enzyme-linked immunosorbent assay (ELISA) kit with a double-antibody sandwich enzyme immunoassay technique [Human Chitinase-3-like protein 1 (YKL-40, CHI3L1)] ELISA Kit, Cat. No. YHB0684Hu; Shanghai Yehua Biological Technology Co. Ltd, China). Each ELISA analysis was carried out according to the manufacturer’s instructions. All tests showed intra- and inter-assay coefficients of variations (CVs) below 8% (n=15) and 10%, respectively. The analytical sensitivity of the test was 0.52 ng/mL.

### Measurement of serum cancer antigen 72-4 concentrations

Serum CA 72-4 concentrations were determined using a commercial ELISA kit with a quantitative competitive enzyme immunoassay technique [CA724 (CA724) BioAssay™ ELISA Kit (Human), Cat. No. 184403; Biomol GmbH; Waidmannstr. 35; 22769 Hamburg; Germany]. Each ELISA analysis was carried out in accordance with the manufacturer’s instructions. All tests showed intra- and inter-assay CVs below 7% (n=15) and 9% (n=15), respectively. The analytical sensitivity of the test was 0.1 ng/mL. Biochemical parameters and tumor markers were analyzed using routine methods with commercial kits and autoanalyzer.

### Statistical Analysis

Statistical analyses were performed using the Statistical Package for the Social Sciences (SPSS) version 16.0 software package (SPSS Inc., Chicago, IL, USA), and clinicopathologic variables, including the categorical data, were analyzed using the chi-square or Fisher’s exact test. Mean values were compared between the groups using the independent Samples t-test and one-way analysis of variance (ANOVA) test. All reported confidence interval values were calculated at the 95% level. A probability (p) value of less than 0.05 was defined as statistically significant.

## Results

**Figure 1:**
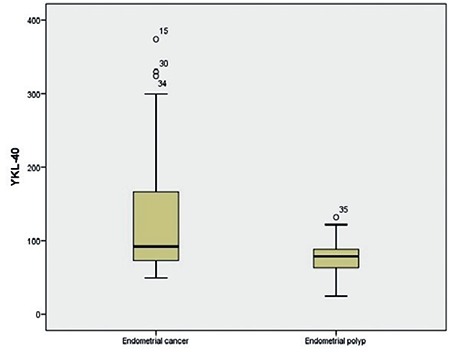
Preoperative serum YKL-40 concentrations in endometrial cancer and endometrial polyps

**Figure 2:**
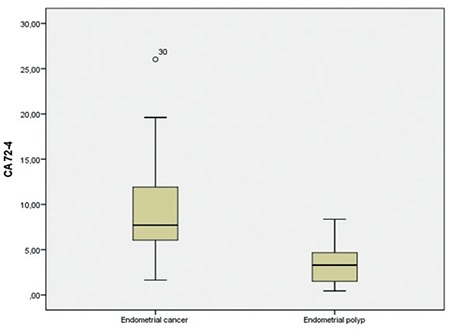
Preoperative serum cancer antigen 72-4 concentrations in endometrial cancer and endometrial polyps CA 72-4: Cancer antigen 72-4

**Figure 3:**
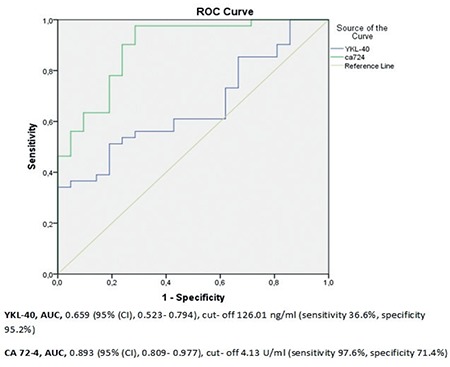
Comparison of ROC curves for YKL-40 and cancer antigen72-4 in the distinction of endometrial polyps from endometrial cancer CA 72-4: Cancer antigen 72-4, CI: Confidence interval

Some of the clinicopathologic data of the study population are summarized in Table 1. The mean age of the subjects in the malignant group was 60.27 (range, 39-82) years and 45.29 (range, 27-70) years in control group. The age of the patients, body mass index, preoperative serum YKL-40 and CA 72-4 levels were significantly higher in the malignant group compared with the control group (Figures 1, 2). In all patients, serum YKL-40 and CA72-4 levels were higher in postmenopausal compared with premenopausal patients. YKL-40 and CA 72-4 levels in premenopausal and postmenopausal patients were 88.4±60.4 ng/mL and 129.9±79.3 ng/mL (p=0.023), and 5.9±5.5 U/mL and 8.56±4.6 U/mL (p=0.047), respectively. Among patients with EC, 36 (87.8%) had endometrioid and 5 (12.2%) had serous histopathologic type. The distribution of surgical stages was as follows: 63.4% in stage 1, 22% in stage 2, 9.7% in stage 3, and 4.9% in stage 4. Histologic grades were found as grade 1 in 24.4%, grade 2 in 53.7%, and grade 3 in 21.9% of the patients with EC. Twenty-five and 16 patients underwent laparotomic and laparoscopic staging surgery, respectively. Systematic pelvic lymphadenectomy was performed in all patients, and para-aortic lymphadenectomy was performed in 11 (27%) patients. The median numbers of pelvic and para-aortic lymph nodes removed were 20.48±9.4 (range, 10-46) and 10.55±6 (range, 6-23), respectively. Postoperative adjuvant treatment was administered to 28 (69%) patients. Intracavitary radiotherapy was given to all patients who needed adjuvant treatment, and external beam radiotherapy was given to 42.8%. Seven patients who had stage 3-4 disease or serous-type cancer also received adjuvant chemotherapy. Older age, advanced stage disease, serous histopathologic type, high grade, deep myometrial invasion, cervical stromal involvement, and lymph node involvement were associated with lower serum YKL-40 levels (Table 2). Serum YKL-40 levels were significantly higher in patients with superficial myometrial invasion and no lymph node involvement (p=0.042; p=0.004). No relationship between clinicopathologic factors and serum CA 72-4 levels was found (Table 2). Receiver operating characteristics curve analyses for YKL-40 and CA 72-4 are shown in Figure 3. The area under the curve was 0.893, and 0.659 for CA 72-4 and YKL-40, respectively. A cut-off value of 4.13 U/mL for CA 72-4 revealed 97.6% sensitivity, 71.4% specificity, 87% positive predictive value (PPV) and 93.8% negative predictive value (NPV). A cut-off value of 126.01 ng/mL for YKL-40 revealed 36.6% sensitivity, 95.2% specificity, 93.8% PPV and 43.5% NPV. The positive likelihood ratiowas 7.6 and 3.4 for YKL-40 and CA 72-4, respectively (Table 3).

## Discussion

The incidence of EC has been on the rise during recent years. Although the majority of cases are diagnosed early, survival rates in advanced stages are likely to be lower. Tumor markers that could be used in early diagnosis of the EC have been investigated in the literature^(23)^. However, there is no evidence for the clinical usefulness of serum tumor markers for routine use in EC screening. CA-125 has been reported to be elevated in 19% to 40% of patients with EC^(2,3)^. Serum HE-4 levels were significantly higher in recurrent EC and superior to CA-125 levels in detecting recurrent EC^(5)^. CA-125 and HE-4 are significantly correlated with histologic grade, stage, lymph node metastases, myometrial invasion, and cervical involvement in EC^(6,8)^. However, CA-125 and HE-4 values are not useful in the diagnosis of early-stage EC. Some studies reported that serum YKL-40 levels were higher in patients with EC compared with healthy individuals^(24,25)^. Fan et al.^(24)^ suggested that, serum YKL-40 had advantages over CA-125 in the diagnosis of early-stage EC, contributing to early management of the disease. Serum YKL-40 levels are also associated with early stage^(24)^. Diefenbach et al.^(26) ^found no statistically significant association of YKL-40 with patient age, tumor grade, histology or stage. The authors claimed that this finding was helpful in the identification of high-risk subsets of patients with worse clinical outcomes^(26)^. In another study, there was no significant difference in terms of the stage and grade of the tumor and for prognostic factors between malignant and benign groups^(27)^. However, YKL-40 was significantly higher in non-endometrioid-type cancer than in endometrioid-type. A recent meta-analysis by Cheng et al.^(28) ^evaluated 234 patients with EC and 300 controls. This meta-analysis concluded that YKL-40 had a moderately high diagnostic accuracy, with a sensitivity of 0.74, a specificity of 0.87 and on the basis of their meta-analysis, therefore, circulating YKL-40 could be promising and meaningful in the diagnosis of EC. In the present study, preoperative serum YKL-40 levels were significantly higher in the malignant group compared with the benign group. Preoperative serum YKL-40 levels were found to be lower in patients with older age, advanced stage, serous type, high grade, deep myometrial invasion, cervical stromal involvement, and lymph node involvement. Serum YKL-40 levels were significantly higher in patients with superficial myometrial invasion and no lymph node involvement (p=0.042). Preoperative serum YKL-40 levels may be associated with favorable prognostic factors. CA72-4 is commonly used as a tumor marker for diagnosing and predicting outcomes in gastric and ovarian cancers^(18,19)^. Anastasi et al.^(29)^ evaluated the CA72-4 values among patients with ovarian cancer (71.0%) and patients with endometriosis (13.8%). They concluded that CA72-4 determination could be useful to confirm the benign nature of ovarian endometriomas in women with high CA-125 levels^(29)^. Serum CA72-4 levels were significantly related with cancer cell lymph node metastasis in pancreatic and gastric cancers^(30,31)^. There are a limited number of studies about the role of CA 72-4 in the diagnosis and prognosis of EC. Regarding this issue, Gadducci et al.^(32)^ demonstrated that serum CA 72-4 levels were raised in approximately 22-32% of the cases in patients with EC. In contrast, Moore et al.^(33) ^found that when compared with control levels, there was no statistically significant difference when comparing serum CA 72-4 levels in all EC stages combined or in stage 1 cancers alone. Soper et al.^(34)^ found that, CA 72-4 levels were elevated (higher than 6 U/mL) in 4% of patients with localized disease and 30% with metastasis. Hareyama et al.^(35)^ reported that serum CA72-4 was increased above the cut-off value in 31.9% of patients with EC. They also found that serum CA72-4 positivity (values >4 IU/mL) was correlated with depth of myometrial invasion, adnexal metastasis, lymphovascular space involvement, and pelvic and para-aortic lymph node metastasis. Hareyama et al.^(35) ^suggested that measuring serum concentrations of CA 72-4 could be useful for predicting and monitoring the progression of disease. Myriokefalitaki et al.^(36)^ investigated the potential additional prognostic benefit of preoperative CA 72-4 level in 282 patients with EC. In this retrospective study, they found that increased CA 72-4 values were statistically significantly correlated with advancing disease stage, which was shorter disease-free survival and higher recurrence rate, hence CA 72-4 appears to be reliable predictor of poor prognosis in patients with EC^(36)^. In our study, preoperative serum CA 72-4 levels were significantly higher in malignant group, compared to benign group. No relationship was found between clinicopathologic factors and serum CA 72-4 levels. Serum CA 72-4 levels had a sensitivity of 97.6%, which demonstrates its sufficiency to distinguish endometrial polyps from EC. The limitations of this study are the low number of patients in the study and control groups.

## Conclusion

The results obtained from our study suggest that measurements of CA 72-4 levels can help differentiate EC from endometrial polyps. Preoperative levels of CA 72-4 can be used as a marker in the early diagnosis of EC. Preoperative serum YKL-40 levels may be associated with favorable prognostic factors. Further prospective studies using large populations and randomized clinical trials are needed to clarify the impact of YKL-40 and CA 72-4 on the definitive diagnosis and prognosis of EC and, eventually, to distinguish benign and malignant endometrial tumors.

## Figures and Tables

**Table 1:**
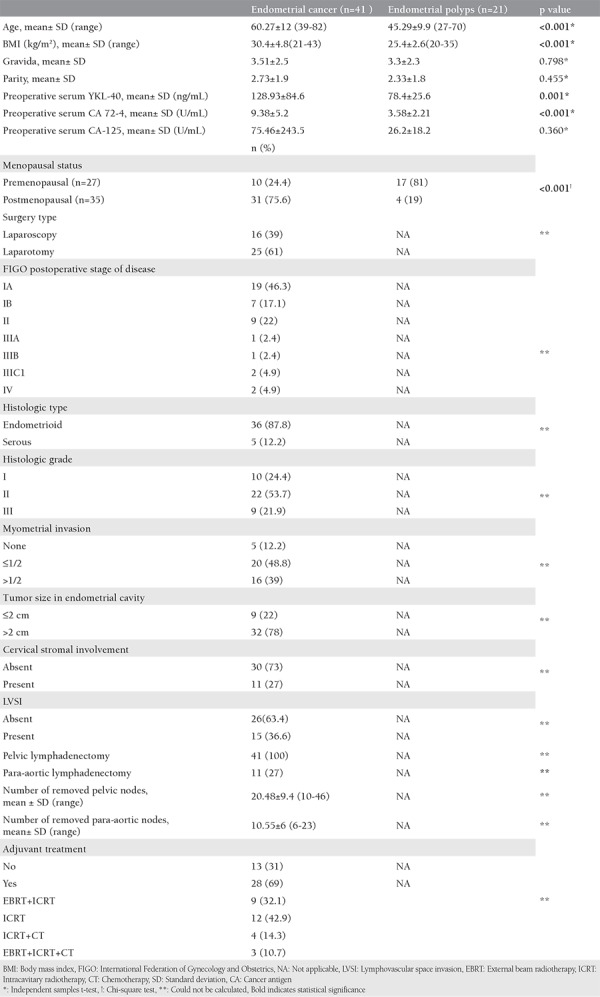
Clinical features and postoperative histopathologic findings of all patients (n=62)

**Table 2:**
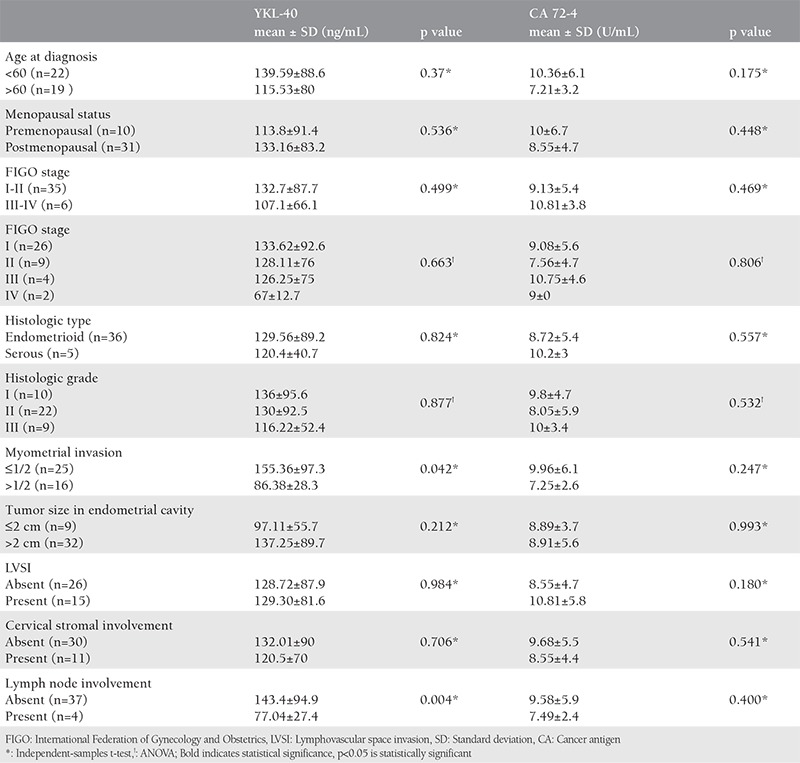
The relationship between YKL-40 and cancer antigen 72-4 results with clinicopathologic factors in the endometrial cancer group (n=41)

**Table 3:**
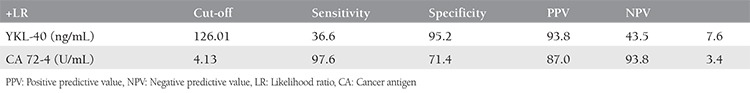
Sensitivity and specificity of YKL-40 and cancer antigen 72-4 in the study groups
